# Supplementary material for: Fragmentation in spin ice from magnetic charge injection
Source: Nat Commun. 2017 Aug 8;8:209. doi: 10.1038/s41467-017-00277-1 (PMC5547148; doi:10.1038/s41467-017-00277-1)
Supplement: Supplementary file 1 — Supplementary information [file 41467_2017_277_MOESM1_ESM.pdf]

File Name: Supplementary Information
Description: Supplementary Figures, Supplementary Notes and Supplementary References

File Name: Peer Review File
Description:

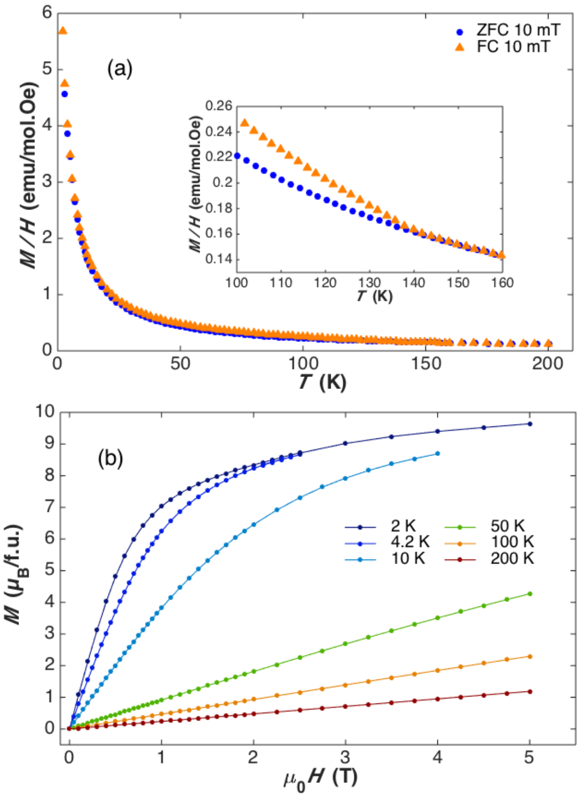


**Supplementary Figure** 1. **Magnetization measurements above 2 K in $Ho_2Ir_2O_7$.** (a) $M/H$ vs $T$ measured after a ZFC (in blue) and a FC (in orange) procedure in 0.01 T. Inset: zoom of the ZFC-FC opening occurring around 140 K. (b)$M$ vs $H$ measured at different temperatures.

## Supplementary Note 1. Inelastic neutron scattering measurements.

On the IN6 cold neutron time-of-flight spectrometer, no signal is detected at the lowest temperature $T = 1.5$ K in the accessible range of energy transfer ($\omega < 2$ meV). Non dispersive signals become visible in the neutron energy gain channel when the temperature is increased, which were ascribed, in the light of the data obtained on IN4, to transitions between crystalline electric field (CEF) levels of the $Ho^{3+}$ ions.

The scattering function $S(Q, \omega)$ measured at 1.5 K on the IN4 thermal neutron time-of-flight spectrometer, for the accessible scattering vector $\mathbf{Q}$ and energy transfer $\omega$ are displayed in Supplementary Figure 2.

Three main signals are essentially observed, at energy transfer $\omega$ around 21, 25 and 56 meV. Their intensity decreases when $Q$ increases, roughly like the square of the magnetic form factor calculated from the radial component of the wavefunction of the $4f^{10}$ electronic configuration of the $Ho^{3+}$ ions [1]. No dispersion is detected within the experimental accuracy. This suggests on one hand that these signals arise from CEF excitations and on the other hand that the Ir-Ho and Ho-Ho exchange energies that govern the collective magnetic behavior of $Ho_2Ir_2O_7$ at low temperature are smaller than the energy resolution in the experiment. All other signals are ascribed to structural dynamics, being observed at high $Q$ or increasing with $Q$. A weak signal was noticed at $\omega$ around 4-5 meV but a careful examination of its $Q$ variation indicated that it cannot be ascribed to a CEF excitation. It can no more be ascribed to a split of the ground doublet by exchange fields. A quantitative estimate indeed shows that this would lead to detectable dispersion on the other excitations and to a thermal variation of the $Ho^{3+}$ magnetic moments very different from the one experimentally deduced from the neutron diffraction experiments.

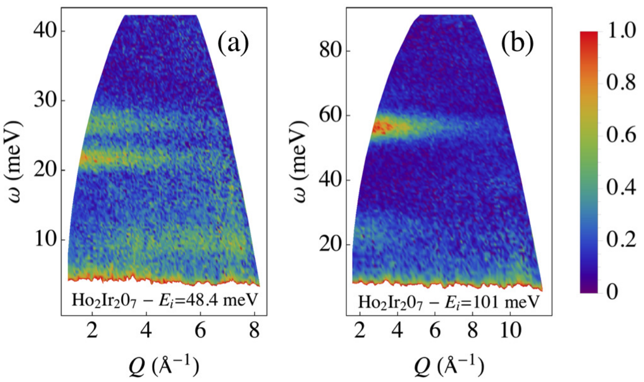


**Supplementary Figure** 2. **Scattering function** $S(Q,\omega)$ **measured at 1.5 K.** The incoming neutron energy is $E_i = 48.4$ meV (a) and $E_i = 101$ meV (b). The data are normalized with respect to the maximum of $S(Q,\omega)$ in the inelastic region ($\omega \geq 1.5\ \delta$E).

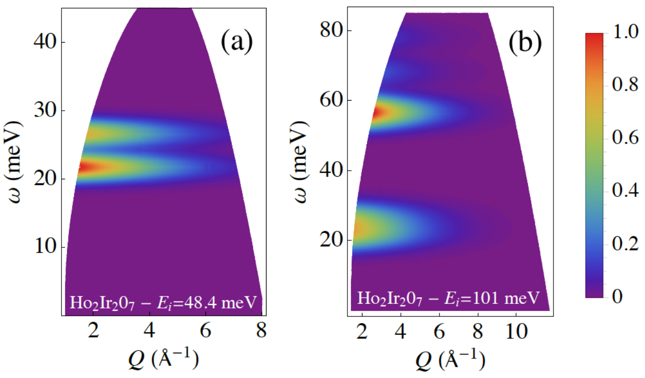


**Supplementary Figure** 3. **Scattering function** $S(Q,\omega)$ **calculated at 1.5 K**. The incoming neutron energy is $E_i = 48.4$ meV (a) and $E_i = 101$ meV (b). $S(Q,\omega)$ is normalized by its maximum.

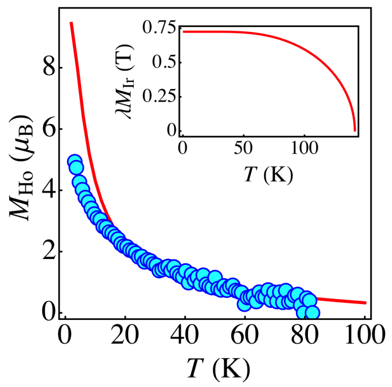


**Supplementary Figure** 4. **Thermal variation of the ordered magnetic moment** $M_{\mathrm{Ho}}$ **induced by the Ir-Ho molecular field.** Cyan disks represent $M_{\mathrm{Ho}}$ obtained from diffraction measurements. The red line corresponds to the calculated moment with the model described in Supplementary Note 2. The inset shows the calculated thermal variation of the Ir-Ho molecular field $\lambda M_{\mathrm{Ir}}(T)$ in Tesla units.

**Supplementary Note 2. CEF analysis and determination of the Ir-Ho molecular exchange field.**

*CEF analysis.* The CEF Hamiltonian for f electrons in the $D_{3d}(\bar{3}m)$ point group symmetry of the 16d site occupied by the $Ho^{3+}$ ions in the $Ho_2Ir_2O_7$ crystal writes

$$\begin{aligned}\mathcal{H}_{\rm CEF} = & B_2^0\mathbf{C}_2^0 + \\ & B_4^0\mathbf{C}_4^0 + B_4^3(\mathbf{C}_4^3 - \mathbf{C}_4^{-3}) + \\ & B_6^0\mathbf{C}_6^0 + B_6^3(\mathbf{C}_6^3 - \mathbf{C}_6^{-3}) + B_6^6(\mathbf{C}_6^6 - \mathbf{C}_6^{-6})\end{aligned} \tag{1}$$

when the quantization axis is chosen along the local 3-fold axis. The $\mathbf{C}_k^q$ stand for Wybourne operators that, in a spatial rotation, transform like the spherical harmonics $Y_k^q$. The $B_k^q$ are the (real) CEF parameters. They can be extracted from the experimentally recorded INS spectra by first diagonalizing $\mathcal{H}_{\rm CEF}$ on the ground multiplet $^5I_8$ ($L = 6$, $S = 2$, $J = 8$) of the $Ho^{3+}$ ions, then by calculating from the obtained eigenvalues $\epsilon_n$ and eigenstates $|n\rangle$ the orientation averaged scattering function

$$S(Q,\omega) = f(Q)^2 \sum_{n,m} \frac{e^{-\beta\epsilon_n}}{Z}\frac{2}{3}|\langle n|\mathbf{J}|m\rangle|^2 \mathcal{R}(\omega - \omega_{n\to m}) \tag{2}$$

where $\beta = \frac{1}{k_{\rm B}T}$ is the temperature factor, $Z = \sum_n e^{-\beta\epsilon_n}$ is the partition function, $\mathbf{J}$ the total angular momentum operator, $\omega_{n\to m} = \epsilon_m - \epsilon_n$ the transition energy from the state $|n\rangle$ to the state $|m\rangle$ and $\mathcal{R}(\omega)$ the convolution of the Dirac $\delta-$function with an instrumental resolution function. The collective nature of the excitations being ignored at this stage, $S(Q,\omega)$ depends on the scattering vector $\mathbf{Q}$ only through the magnetic form factor $f(Q)$. Within the ground multiplet $^5I_8$ this can be computed in the dipole approximation as $f(Q) = \langle j_0(Q)\rangle + (g_L/g_J)\langle j_2(Q)\rangle$ where $g_L = 3/4$ and $g_J = 5/4$ are the standard orbital and total gyromagnetic factors associated with the multiplet $^5I_8$. The radial integrals $\langle j_K(Q)\rangle = \int_0^\infty r^2 R_{\rm 4f}\, j_K(rQ)dr$, where $R_{\rm 4f}$ is the $f^{10}-$electron radial wavefunction and $j_K(rQ)$ the Bessel function of order $K$, are tabulated in the International Tables of Crystallography [1]. The fits of the CEF parameters were carried out by a reverse Monte Carlo method with Metropolis sampling and simulated annealing. The starting parameters were those reported for $Ho_2Ti_2O_7$ [2]. At high temperatures the decay of activated CEF excitations provided additional constraints.

*Ir-Ho molecular exchange field.* Since no dispersion in the crystalline electric field excitations was detected within the accuracy of the experiments, only upper bound for the exchange interactions can be estimated. A physical quantity that however revealed itself strongly sensitive to the Ir-Ho exchange interactions is the ordered magnetic moment $\mathbf{M}_{\rm Ho}$ of the $Ho^{3+}$ ions. This was extracted from the refinement, using an AIAO order for the Ho sublattice, of the neutron diffraction experimental data and can be calculated at all temperatures as

$$\mathbf{M}_{\rm Ho} = g_{\rm J}\mu_{\rm B}\,{\rm Tr}(\mathbf{J}\exp(-\beta(\mathcal{H}_{\rm CEF} + \mathcal{H}_{\rm Ho-Ir}))) \tag{3}$$

by writing the Hamiltonian $\mathcal{H}_{\rm Ho-Ir}$ of the Ir-Ho exchange interactions in the Zeeman form

$$\mathcal{H}_{\rm Ho-Ir} = -\lambda\mathbf{M}_{\rm Ir}(T)\cdot g_{\rm J}\mu_{\rm B}\mathbf{J} \tag{4}$$

where $\lambda$ stands for the Ir-Ho molecular exchange field constant. The thermal variation of the Ir magnetic moment $\mathbf{M}_{\rm Ir}(T)$ is determined self-consistenly in its own Ir-Ir molecular exchange field, by assuming that it can be described as a spin 1/2. The Ir-Ir molecular exchange field constant is determined by imposing that $\mathbf{M}_{\rm Ir}(T)$ cancels self-consistently at the Ir magnetic ordering temperature $T_{\rm MI} \approx 140$ K. A similar processing of exchange interactions was previously implemented in $Tb_2Ir_2O_7$ [3].

*Refined parameters.* The best fits of the INS spectra at the different temperatures and for the different incoming neutron energies, on one hand, and of the thermal variation of the magnetic moments $\mathbf{M}_{\rm Ho}$, on the other hand, were obtained with CEF parameters $B_2^0 \approx 64$ meV, $B_4^0 \approx 242$ meV, $B_4^3 \approx 71$ meV, $B_6^0 \approx 89$ meV, $B_6^3 \approx -83$ meV, $B_6^6 \approx 90$ meV and a Ho-Ir molecular field $\lambda M_{\rm Ir}(0) = 0.725(\pm 0.050)$ T. The scattering function $S(Q,\omega)$ in the neutron energy loss channel calculated at 1.5 K for incoming neutron energies $E_i = 48.4$ meV and $E_i = 101$ meV is displayed in the top panel of Supplementary Figure 3.

The three main experimental signals are reproduced at the awaited energy transfer $\omega$ mimicking their decrease as a function of the scattering vector $Q$. Their relative spectral weights differ to some extent from those of the experimental signals because of contributions from structural dynamics in the experiments. Additional weak signals are visible in the calculated spectra for incoming neutron energy $E_i = 101$ meV that accounts for CEF excitations at higher energy. The CEF leads to a ground doublet well separated from the excited levels that gives rise to a pseudo-Ising magnetic moment of magnitude $\approx$9.6 $\mu_{\rm B}$ oriented along the local three fold axis as in the Ho pyrochlore stannate and titanate [4]. The parallel ($\|$) and perpendicular ($\perp$) Landé factors are calculated to $g_\| = 19.177$ and $g_\perp = 0.002$.

The calculated thermal variation of the magnetic moment $M_{\mathrm{Ho}}$ of the $\mathrm{Ho}^{3+}$ ions induced by the Ho-Ir exchange field is displayed in the bottom panel of Supplementary Figure 4 and compared to the experimental values extracted from the neutron diffraction data (see main article). They disagree below 20 K. No fitting of the whole experimental variation can be performed with only the Ho-Ir exchange field. As a matter of fact the temperature at which the measured and calculated moments depart from each other announces the onset of the magnetic correlations associated with the exchange interaction between the $\mathrm{Ho}^{3+}$ moments and the beginning of the fragmentation process.

## Supplementary Note 3. Low temperature AC susceptibility

AC susceptibility measurements in $\mathrm{Ho_2Ir_2O_7}$ were performed at a fixed frequency $f$, as a function of temperature $T$ (as shown in the main text in Figure 3), and at a fixed temperature, as a function of frequency (see Supplementary Figure 5). The out-of-phase susceptibility $\chi''$ presents a maximum in both measurements, which is the signature of the presence of energy barriers that the system has to overcome when it is subjected to a magnetic field. Nevertheless, the curves are very broad, suggesting that the energy barrier is not unique, and that a distribution exists. In particular, the $\chi''$ vs $f$ curve does not display the lorentzian shape which is expected in presence of a single energy barrier. While at intermediate temperatures (typically $500-750$ mK), the curves can be fitted with a gaussian centered at a single frequency, at lower and higher temperatures, the shape is very asymmetric, indicating a distribution as well as several characteristic energies. For the lowest temperatures, due to the very long relaxation times, the system might have not reached its thermodynamic equilibrium, so that the energy distribution we have probed may be related to out-of-equilibrium processes.

For these reasons, the maxima obtained from $\chi''$ vs $f$ and $\chi''$ vs $T$ measurements do not match, especially in the low and high temperature ranges (see Supplementary Figure 6), giving rise to different relaxation times $\tau$ (such discrepancy had already been reported in the spin ice $\mathrm{Ho_2Ti_2O_7}$ [5]). Note that no relaxation time could be determined below 350 mK and above 1.1 K in $\chi''$ vs $f$ curves due to the broad shape of the curves. The true relaxation time, defined in the context of Debye relaxation, is the one obtained from the $\chi''$ vs $f$ curves and it follows an Arrhenius law, as described in the main text.

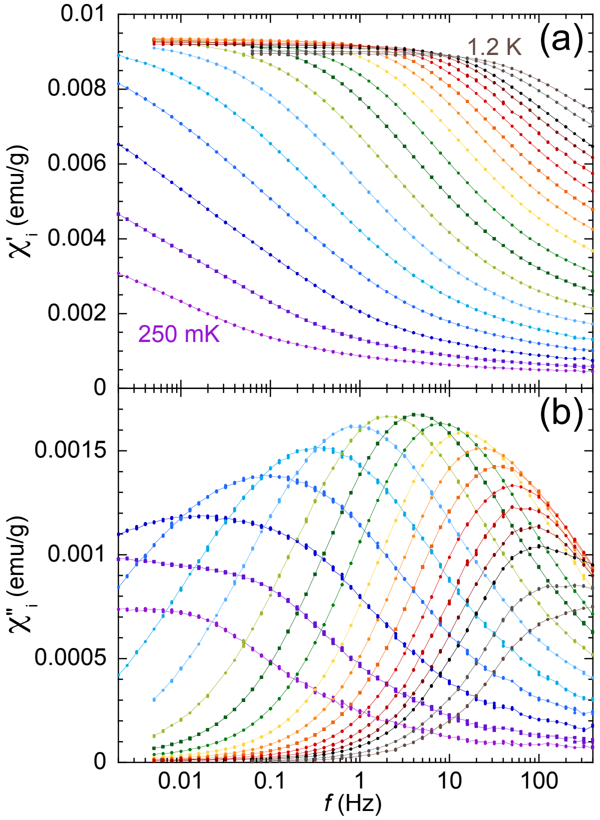


**Supplementary Figure 5. AC susceptibility as a function of frequency.** (a) $\chi'_{\mathrm{i}}$ and (b) $\chi''_{\mathrm{i}}$ are corrected from demagnetizing effects, and were measured between 250 mK and 1.2 K (by steps of 50 mK up to 1 K, and 100 mK above), with $\mu_0 H_{\mathrm{AC}} = 0.1$ mT.

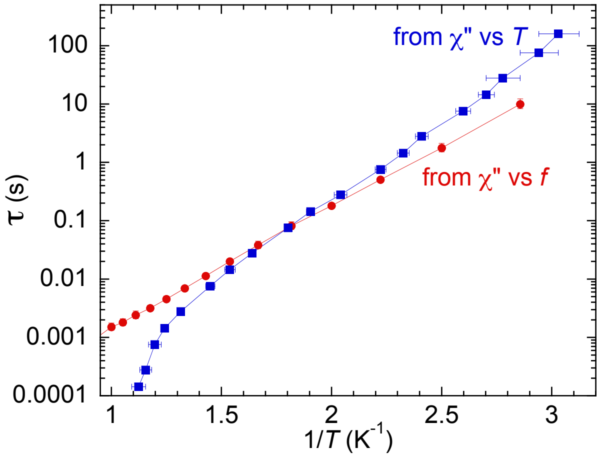


**Supplementary Figure** 6. **Relaxation time** $\tau = 1/2\pi f$ **vs** $1/T$. $\tau$ was determined from the temperature of the $\chi''$ maximum measured at constant frequency (blue squares) and from the frequency of the $\chi''$ maximum measured at constant temperature (red circles). The error bars correspond to the error in the determination of the positions of the maxima.

## Supplementary Note 4. Theoretical description of the dynamics

*Charge model.* Below, we present the mapping of the spin model into a magnetic charge model, providing an alternative description of the local excitations (defects) and an insight into the origin of the energy barrier observed in AC susceptibility.

Defining the charge $q_\alpha = \frac{\Delta_\alpha}{2} \sum_{i\in\alpha} \sigma_i$ on the tetrahedron $\alpha$, with $\Delta_\alpha = +1, -1$ for in and out tetrahedra respectively, the following spin Hamiltonian

$$\mathcal{H} = \mathcal{J}_{\text{eff}} \sum_{\langle i,j \rangle} \sigma_i \sigma_j - h_{\text{loc}} \sum_i \sigma_i, \tag{5}$$

defined in the main text, can be written

$$\mathcal{H} = \sum_\alpha 2\mathcal{J}_{\text{eff}} q_\alpha^2 - h_{\text{loc}} \Delta_\alpha q_\alpha - 2\mathcal{J}_{\text{eff}}. \tag{6}$$

From Supplementary Equations 5-6, the different spin configurations of a given tetrahedron have the following energies (displayed on Supplementary Figure 7(a) as a function of the local field $h_{\text{loc}}/\mathcal{J}_{\text{eff}}$):

$$\begin{array}{lllll} E_{\text{2I2O}} & = E_{q=0} & = -2\mathcal{J}_{\text{eff}} & & \\ E_{\text{3I1O}-\text{1I3O}} & = E_{q=\pm1} & = & \mp & \Delta_\alpha h_{\text{loc}} \\ E_{\text{4I0O}-\text{0I4O}} & = E_{q=\pm2} & = 6\mathcal{J}_{\text{eff}} & \mp & 2\Delta_\alpha h_{\text{loc}} \end{array} \tag{7}$$

Note that since each site is shared by two tetrahedra, so is the local field: the field term of Supplementary Equations 7 is then divided by a factor 2. This finally leads to the two phase boundaries, $h_{\text{loc}}/\mathcal{J}_{\text{eff}} = 2$ ($E_{q=0} = E_{q=\pm1}$) separating the 2I2O (spin ice) from the 3I1O–1I3O (fragmented) ground state, and $h_{\text{loc}}/\mathcal{J}_{\text{eff}} = 6$ ($E_{q=\pm1} = E_{q=\pm2}$) separating the 3I1O–1I3O (fragmented) from the 4I0O–0I4O (ordered) ground state.

These phase boundaries are confirmed by Monte Carlo simulations performed on extended pyrochlore lattices for different local fields $h_{\text{loc}}/\mathcal{J}_{\text{eff}}$ and reduced temperatures $T/\mathcal{J}_{\text{eff}}$, as shown in Supplementary Figure 7(b) (as well as the Figure 1(c) of the main text).

*Propagation of defects in the fragmented regime.* In spin ice materials, the diffusion of monopoles causes a slow relaxation of the magnetization which can be probed experimentally (see Supplementary Note 3 and main text). As a consequence, the relaxation timescale is proportional to the mobility of monopoles and inversely proportional to their density [6–8]: the more defects in the system, the faster the relaxation.

In the fragmented regime ($2 < h_{\text{loc}}/\mathcal{J}_{\text{eff}} < 6$), the elementary excitations, although still fractional, are not canonical monopoles anymore: flipping a spin from the 3I1O–1I3O ground state now creates a pair of 2I2O (or 4I0O–0I4O)

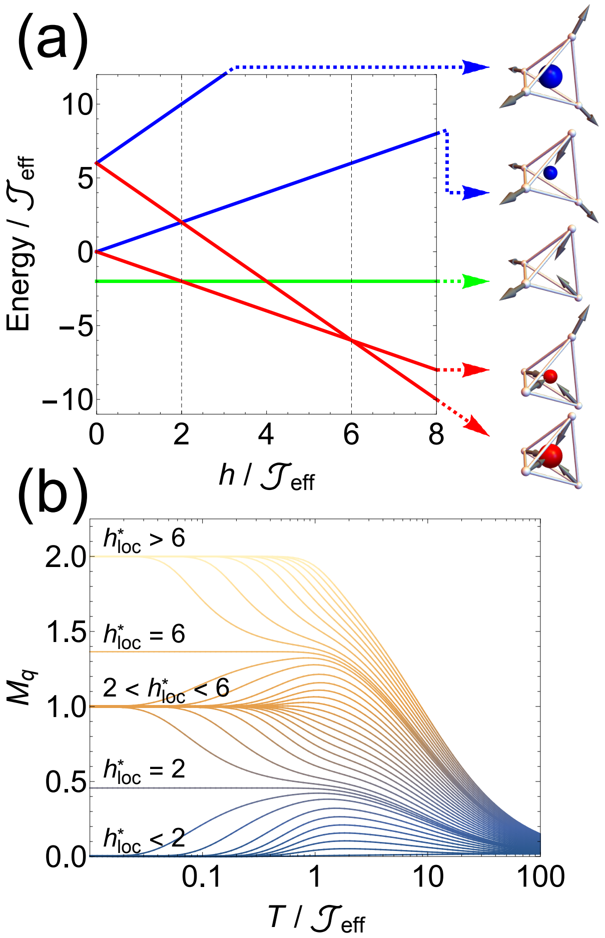


**Supplementary Figure 7. Energies of the spin configurations and calculated order parameter.** (a) Energies, in $\mathcal{J}_{\rm eff}$ units, of the different spin configurations of a single in tetrahedron as a function of the local field $h^*_{\rm loc} = h_{\rm loc}/\mathcal{J}_{\rm eff}$, showing the succession, when increasing the field, of the three different ground states 2I2O, 3I1O, and 4I0O. The red, blue and green colors indicate a positive, a negative and zero charge on a tetrahedron respectively. The small (resp. large) spheres represent charges $q = \pm 1$ (resp. $q = \pm 2$). (b) Charge order parameter $M_q$ (defined in the main text) as a function of the temperature $T/\mathcal{J}_{\rm eff}$ for different local fields $h^*_{\rm loc}$.

defects [9]. We show in the following that the dynamics of these defects, propagating through different processes which depend on the value of $h_{\rm loc}$, is strongly unusual.

*Single excitation process.* We first focus on the most simple mechanism, dominant for $3 < h_{\rm loc}/\mathcal{J}_{\rm eff} < 5$, i.e. deep inside the fragmented regime, and which has been shortly discussed in the main text. It is schematized, on Supplementary Figure 8(a), by the motion of a single defect along an unfolded string of tetrahedra. Its peculiarity comes from two (related) points:
(i) moving one defect from a tetrahedron to its neighbor by flipping one spin changes the nature of the defect: a 2I2O excitation transforms into a 4I0O (or 0I4O) excitations and vice versa, so that its propagation involves two alternating excitations.
(ii) the energies of the 2I2O and 4I0O–0I4O excitations, which depend on the local field $h_{\rm loc}$, are generally different (except for $h_{\rm loc} = 4\mathcal{J}_{\rm eff}$) (see Supplementary Figure 7(a)), and so are their densities.

Interestingly, mapping this spin picture onto a charge description gives a better understanding of the dynamical properties of the fragmented regime, by characterizing the explicit role and behavior of each fragment: the defects are, in such a description, the elementary excitations (charges) of the Coulomb phase (divergence-free fragment), propagating in the periodic potential induced by the underlying static charge crystal (divergence-full fragment).

To show this, let us first consider that the charge $q_\alpha$ of the tetrahedron $\alpha$ is the combination of a static charge $q^\alpha_o = \Delta_\alpha$ (ordered charge of the charge crystal), and of a propagating charge $q$ (elementary excitation of the Coulomb phase). The $q$ value depends on the presence ($q = \pm 1$) or not ($q = 0$) of a defect on the tetrahedron $\alpha$. The energy

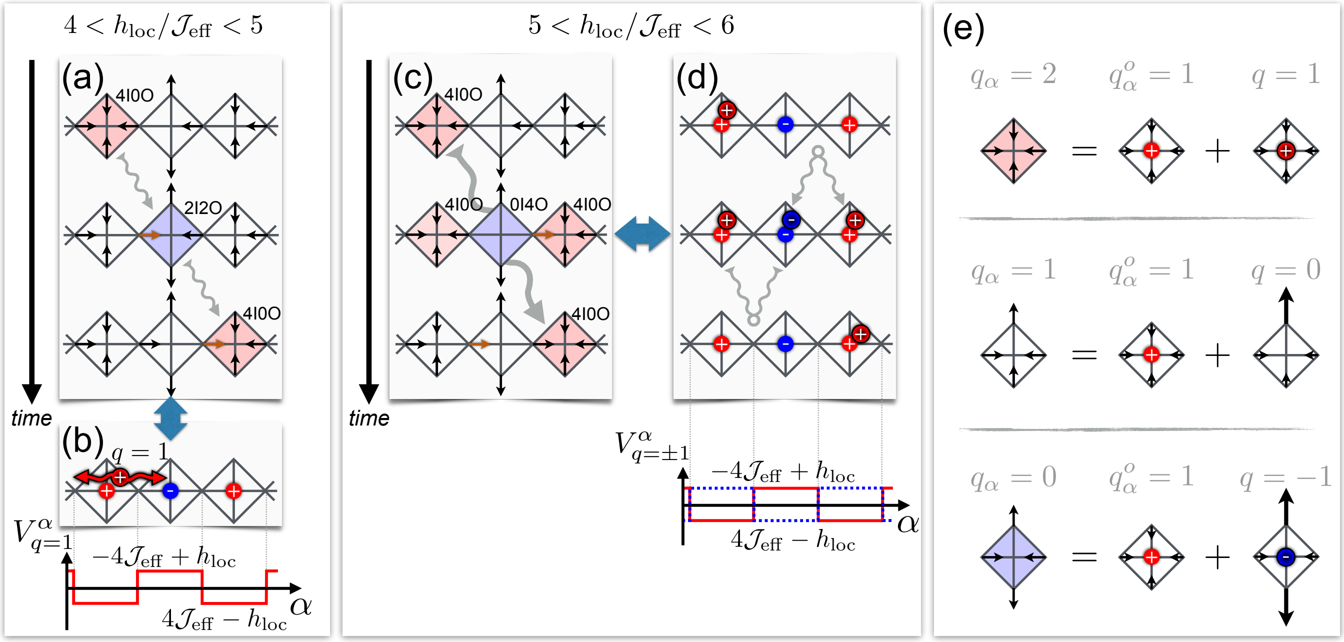


**Supplementary Figure** 8. **Diffusion mechanisms in the spin and charge pictures.** (a-b) Direct diffusion mechanism of a single defect carrying a positive charge $q = 1$, dominant for $3 < h_{\rm loc}/\mathcal{J}_{\rm eff} < 5$, using spin (a) and charge (b) representations. The oscillating gray arrows show the motion of the defect at arbitrary times. The periodic potential $V^{\alpha}_{q=1}$ is shown for $4 < h_{\rm loc}/\mathcal{J}_{\rm eff} < 5$, and would be the opposite for $3 < h_{\rm loc}/\mathcal{J}_{\rm eff} < 4$. (c-d) Indirect diffusion mechanism of a single defect carrying a positive charge $q = 1$, dominant for $5 < h_{\rm loc}/\mathcal{J}_{\rm eff} < 6$, using spin (c) and charge (d) representations. The gray arrows show the spontaneous creation ($t = 1$) and annihilation ($t = 2$) of two defects with opposite charges $q = \pm1$. At bottom, the dashed blue line (resp. plain red line) represents the periodic potential of a negative (resp. positive) charge. The same mechanism occurs for $2 < h_{\rm loc}/\mathcal{J}_{\rm eff} < 3$ for 2I2O defects. (e) Mapping between the spin and the charge pictures for an in tetrahedron.

of such a defect is obtained by injecting $q_\alpha = q^{\alpha}_o + q$ in the Hamiltonian (6), and writes

$$E^{\alpha}_q = 2\mathcal{J}_{\rm eff} + V^{\alpha}_q, \tag{8}$$

with the required energy to create a free charge $2\mathcal{J}_{\rm eff}$, and the potential energy $V^{\alpha}_q = 4\mathcal{J}_{\rm eff} q^{\alpha}_o q - h_{\rm loc}\Delta_\alpha q$. Noting that, in the fragmented regime, $q^{\alpha}_o = \Delta_\alpha$, one can obtain the more simple expression

$$V^{\alpha}_q = (4\mathcal{J}_{\rm eff} - h_{\rm loc})\, q^{\alpha}_o\, q$$

which takes the form of an on-site interaction periodic potential induced by the underlying charge crystal, and is represented in Supplementary Figure 8(b) for a defect carrying a positive charge $q = +1$. For $h_{\rm loc}/\mathcal{J}_{\rm eff} > 4$, charges of opposite (resp. similar) sign are repulsive (resp. attractive), while the opposite situation is realized for $h_{\rm loc}/\mathcal{J}_{\rm eff} < 4$.

Coming back to a spin description of the dynamics, as shown in Supplementary Figure 8(e), this means that the 2I2O defects (with a total charge $q^{\alpha}_o + q = 0$) have a lower energy than the 4I0O–0I4O ones ($q^{\alpha}_o + q = \pm2$) for $h_{\rm loc}/\mathcal{J}_{\rm eff} > 4$, and the opposite situation for $h_{\rm loc}/\mathcal{J}_{\rm eff} < 4$ (see Supplementary Figure 7(a)). The relaxation timescale $\tau = \tau_0 \exp(\beta \Delta E_1)$ in this single excitation regime is, finally, the time needed to overcome the energy barrier of the potential well $\Delta E_1 = h_{\rm loc} - 2\mathcal{J}_{\rm eff}$ (resp. $6\mathcal{J}_{\rm eff} - h_{\rm loc}$) for $h_{\rm loc}/\mathcal{J}_{\rm eff} > 4$ (resp. $< 4$) (see the cyan lines in Supplementary Figure 9(a)).

*Multi-excitation process.* Approaching the critical fields $h_{\rm loc} = 2\mathcal{J}_{\rm eff}$ and $6\mathcal{J}_{\rm eff}$ in the fragmented state, the charge repulsion becomes such that another (more indirect but energetically favorable) diffusion process comes into play, accelerating the dynamics. As represented in Supplementary Figure 8(c), this mechanism involves two successive spin flips. The first spin flip creates a pair of 2I2O (resp. 0I4O–4I0O) defects on the two neighboring tetrahedra of an already existing 2I2O (resp. 0I4O or 4I0O) defect. Then, the second spin flip simply annihilates one of these two defects with the old one, in such a way that this latter finally moved two tetrahedra away. This mechanism has the advantage to avoid the charge repulsion discussed earlier, by creating (close to an existing charge) two charges of opposite sign which both have an attractive potential with the charge crystal (Supplementary Figure 8(d)).

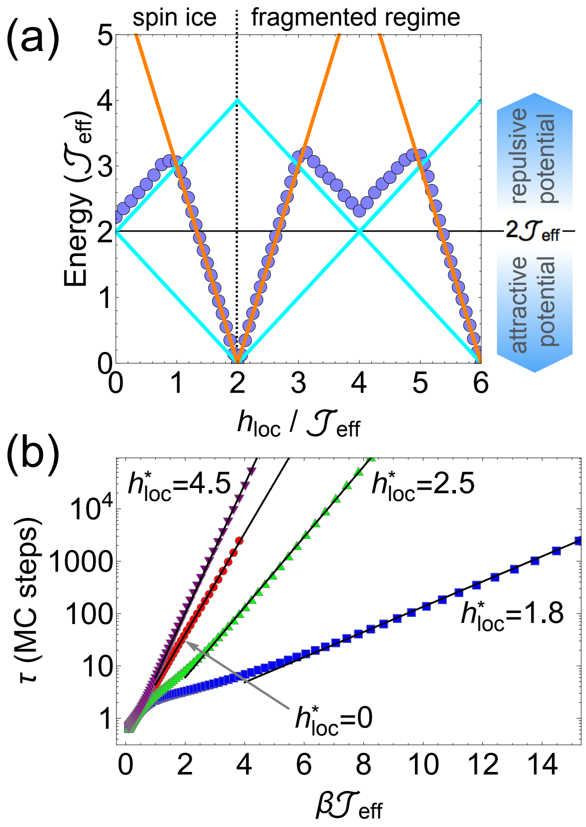


**Supplementary Figure** 9. **Characteristic energies and relaxation timescale in the diffusion processes.** (a) Characteristic energies associated to the energy barrier $\Delta E$ leading to the dynamical properties probed by AC susceptibility and setting the timescale $\tau = \tau_0 \exp(\beta \Delta E)$, as a function of the local field $h_{\rm loc}/\mathcal{J}_{\rm eff}$, obtained by Monte Carlo simulations for $N = 8192$ spins (blue circles). The blue and orange lines represent single and multi-excitation processes respectively and their equations are given by the energies $E^\alpha_q$ of a single defect (Supplementary Equation 8) and by $3E^\alpha_q$. As explained in the text, the single excitation process prevails for $3 < h_{\rm loc}/\mathcal{J}_{\rm eff} < 5$ and the multi-excitation process prevails close to the boundaries with the 2I2O ($2 < h_{\rm loc}/\mathcal{J}_{\rm eff} < 3$) and AIAO ($5 < h_{\rm loc}/\mathcal{J}_{\rm eff} < 6$) phases. (b) Characteristic relaxation timescale $\tau$ as a function of the inverse temperature $\beta$ for different values of the local field $h^*_{\rm loc} = h_{\rm loc}/\mathcal{J}_{\rm eff}$. The symbols result from the fit of the correlation function $c(t)$ at each temperature, while the black lines represent the fit of $\tau$ vs $\beta$ (see text).

The energy cost for such a motion is, therefore, the energy of three charges trapped into their potential well $\Delta E_2 = 18\mathcal{J}_{\rm eff} - 3h_{\rm loc}$ (resp. $3h_{\rm loc} - 6\mathcal{J}_{\rm eff}$) for $h_{\rm loc}/\mathcal{J}_{\rm eff} > 4$ (resp. $< 4$). A direct comparison of $\Delta E_1$ and $\Delta E_2$ shows that the second mechanism, represented by the orange plain line in Supplementary Figure 9(a), is energetically favorable for $h_{\rm loc}/\mathcal{J}_{\rm eff} < 3$ and $> 5$.

*Numerical simulations.* This local field dependence of the charge dynamics has been confirmed by the numerical calculations of the autocorrelation function $c(t) = \frac{1}{N}\sum_i \langle \sigma_i(0)\sigma_i(t)\rangle$ using Monte Carlo simulations, with $t$ the Monte Carlo time, and $N = 8192$ the number of spins. In those simulations, the metropolis single spin flip algorithm, which mimics the local dynamics of the rare earth ions, has been used to evaluate $c(t)$, while it has been combined to a loop algorithm for thermalization. $c(t)$ has been fitted at each temperature and for different values of the local field within the range $2 < h_{\rm loc}/\mathcal{J}_{\rm eff} < 6$, assuming an exponential decrease of the autocorrelation with time $c(t) = \exp(-t/\tau)$. Then, the resulting characteristic timescale has been fitted as a function of the temperature, for $T/\mathcal{J}_{\rm eff} \lesssim 1$ and for each value of the local field, assuming an activation law $\tau = \tau_0 \exp(\beta \Delta E)$. This procedure, shown in Supplementary Figure 9(b) for several local fields, provides a numerical evaluation of the energy barrier $\Delta E$. The numerical simulations are in good agreement with the predictions discussed above, although the energy barriers obtained numerically for the single excitation process are slightly larger than expected.

Note also that the second mechanism is observed only at temperatures significantly lower than the energy difference between the two closest ground states (2I2O and 3I1O–1I3O around $h_{\rm loc}/\mathcal{J}_{\rm eff} = 2$, and 3I1O–1I3O and 4I0O–0I4O around $h_{\rm loc}/\mathcal{J}_{\rm eff} = 6$). At larger temperatures, these two spin configurations have similar Boltzmann weights and can coexist without a significant energy cost. Simple spin flips then allow the system to fluctuate between the two

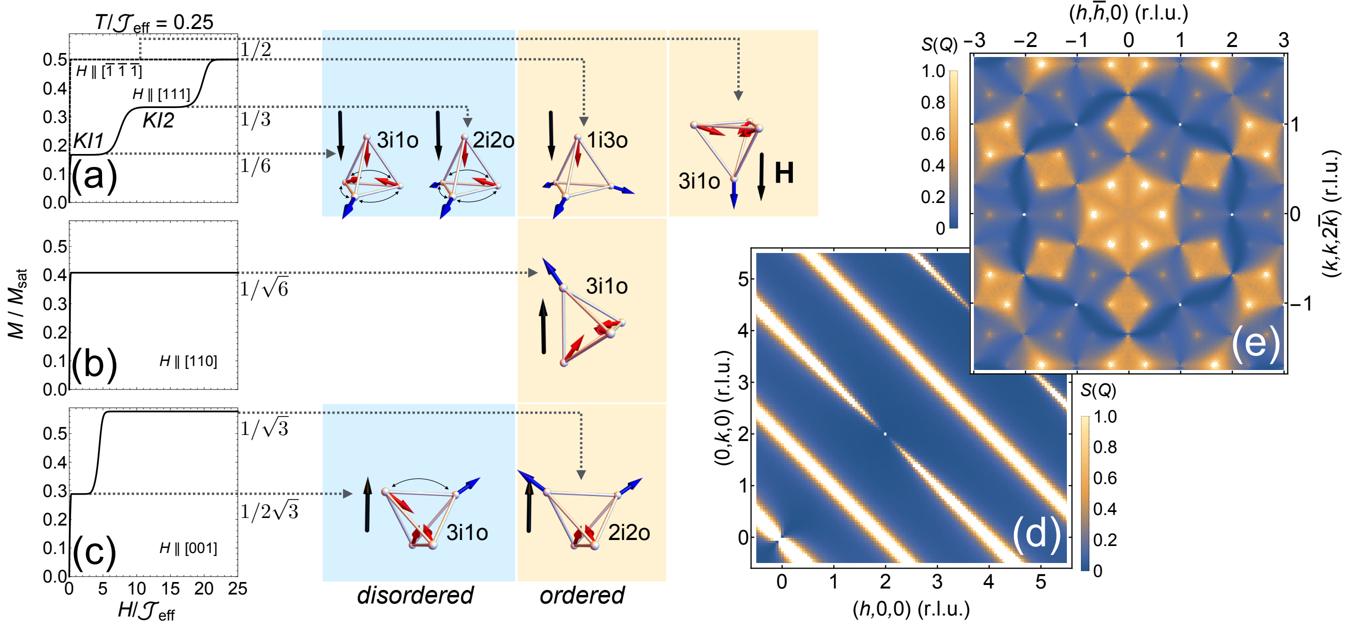


**Supplementary Figure 10. Field induced magnetic behavior.** (a-c) Magnetization $M$ per spin (assuming $S = 1$), obtained from Monte Carlo simulations for $N = 8192$ spins, as a function of the global field $H$ applied (a) along the [111] and $[\bar{1}\bar{1}\bar{1}]$ directions (plain and dashed curves), (b) along the [110] direction, and (c) along the [001] direction. The value of the magnetization on each plateau is indicated on the right of the plots, together with a scheme of the stabilized spin configurations considering an in tetrahedron. Note that inverting the field from [111] to $[\bar{1}\bar{1}\bar{1}]$ is equivalent to going from one AIAO iridium 180° domain to the other one. KI1 and KI2 label the successive fragmented kagome ice phases stabilized for $\mathbf{H} \parallel [\bar{1}\bar{1}\bar{1}]$. Their scattering function, exhibiting Bragg peaks and pinch point pattern, is represented in (e). The scattering function of the partially disordered state stabilized for $\mathbf{H} \parallel$ [001], shown in (d), displays rods of diffuse scattering.

configurations. Note finally that the case $h_{\rm loc}/\mathcal{J}_{\rm eff} < 2$, supporting a 2I2O Coulomb phase, has not been discussed above, although similar dynamics occur. The main difference with the fragmented regime is the absence of charge order, such that the periodic potential is here only created by the staggered local field $V_q^\alpha = -h_{\rm loc}\Delta_\alpha q$ which plays a role comparable to the charge crystal in the fragmented regime.

## Supplementary Note 5. Magnetization in the presence of an external field

As noted in the main text, the measured magnetization of $Ho_2Ir_2O_7$ in an external magnetic field $\mathbf{H}$ presents an unconventional behavior. Due to the powder average, these data are difficult to analyze quantitatively. However, we anticipated that rich plateau physics should underlie our observations. We have thus performed Monte-Carlo calculations for $h_{\rm loc}/\mathcal{J}_{\rm eff} = 4.5$, the value we have estimated for $Ho_2Ir_2O_7$, to get a first insight into the involved physics. These calculations indeed disclose the stabilization of field-induced exotic phases.

When the field is applied along the [111] direction, for which the pyrochlore lattice consists of alternating kagome and triangular planes, we get two successive magnetization plateaus KI1 and KI2 (see Supplementary Figure 10(a)). They correspond to kagome ice phases, i.e. fragmented phases in the kagome planes supporting a 2I1O–1I2O charge crystal together with fluctuating disordered spins, while the spins of the triangular planes are aligned along the magnetic field. The corresponding scattering function results in the coexistence of a diffuse signal presenting pinch points and of Bragg peaks associated with both the charge order in the kagome planes with a propagation vector $\mathbf{k} = (2/3, 2/3, 0)$ and the polarized spins of the triangular lattice with $\mathbf{k} = \mathbf{0}$ [10] (see Supplementary Figure 10(e)).

Although KI1 and KI2 stabilize the same charge order in the kagome planes, they support distinct three dimensional charge states, with different rules on each tetrahedron and different magnetization values. Those different spin configurations reflect the competition between the staggered field $h_{\rm loc}$ and the increasing external field $H$. When $H$ is small (KI1), the field-induced state on the first plateau still corresponds to a charge crystal on the pyrochlore lattice, made of 3I1O and 1I3O configurations: the spins in the triangular planes are polarized by the applied field, and the kagome ice forms in the kagome planes with one spin having a positive projection along the field and the two others a negative one, so that the global rules 3I1O (or 1I3O) on the tetrahedra are preserved (see Supplementary Figure 10(a)

for an in tetrahedron). This results in a magnetization plateau at $M = \dfrac{1}{N}\mathbf{H}\cdot\left(\sum_i \mathbf{S}_i\right) = \frac{1}{4}\left(1+\frac{1}{3}-\frac{1}{3}-\frac{1}{3}\right) = \frac{1}{6}$ with $N$ the number of spins and assuming $S=1$.

When $H$ increases (KI2), the Zeeman energy is such that it becomes favorable, in each triangle of the kagome plane, to flip a spin so that its projection along the field goes from negative to positive. Each tetrahedron then stabilizes a 2I2O spin configuration, still inducing a kagome ice state, but leading to a global jump in the magnetization curve towards a plateau at $M = \frac{1}{4}\left(1+\frac{1}{3}+\frac{1}{3}-\frac{1}{3}\right) = \frac{1}{3}$. Note that the phase KI2 is the same as in a conventional spin ice under a [111] applied field [11, 12]. KI1 and KI2, having exactly the same correlations in the kagome planes, present the same scattering function as well (identical diffuse scattering and Bragg peaks of the charge crystal). Actually, they only differ by the intensity of their Bragg peaks at Brillouin zone centers.

At larger field, the system further polarizes in the $\mathbf{H}$ direction leading to an ordered 3I1O–1I3O state with $M = \frac{1}{4}\left(1+\frac{1}{3}+\frac{1}{3}+\frac{1}{3}\right) = \frac{1}{2}$. This plateau succession only concerns one out of the two possible 180° spin domains, themselves selected by the underlying 180° magnetic domains of the AIAO iridium magnetic order. The second domain − which can be obtained by reversing all iridium spins and thus all the local fields − is immediately polarized in a 1I3O–3I1O ordered phase when a [111] external field is applied, as shown by the dashed curve in Supplementary Figure 10(a). Note that inverting the local fields or the global applied field produces the same effect, so that this dashed curve has been labelled $\mathbf{H} \parallel [\bar{1}\bar{1}\bar{1}]$.

For a field applied along the [001] direction, a magnetization plateau is also observed at half the saturation $M = \frac{1}{4\sqrt{3}}(1+1+1-1) = \frac{1}{2\sqrt{3}}$, associated with long range 1D correlations along the spin chains perpendicular to the field. These spin chains can be separated into two sets: one, fully ordered, has all its spins preferentially oriented in the field direction (chains along $[1\bar{1}0]$ in Supplementary Figure 10(b)); the second set, characterized by alternating in and out spins along the [110] chains, remains disordered from chain to chain. Therefore, the resulting scattering function in the $(h,k,0)$ scattering planes shows rods of diffuse scattering along $[1\bar{1}0]$ together with Bragg peaks at Brillouin zone centers (See Supplementary Figure 10(d)). Note that this state is similar to the spin ice case with a field applied along the [110] direction [13, 14].

Finally, for a field applied along the [110] direction, the magnetization saturates very fast toward a 3I1O–1I3O ordered state with $M = \frac{1}{\sqrt{6}}$ (Supplementary Figure 10(c)).


[1] Anderson, I. S., Carpenter, J. M., Lander, G., Pynn R., Rowe J. M., Schärpf, O., Sears, V. F., and Willis, B. T. M. *International Tables for Crystallography*, ch 4.4: Neutron techniques, vol. C (2006).

[2] Ruminy, M., Pomjakushina, E., Iida, K., Kamazawa, K., Adroja, D. T., Stuhr, U., and Fennell, T. Crystal-field parameters of the rare-earth pyrochlores $R_2Ti_2O_7$ (R=Tb, Dy, and Ho). *Phys. Rev. B* **94**, 024430 (2016).

[3] Lefrançois, E., Simonet, V., Ballou, R., Lhotel, E., Hadj-Azzem, A., Kodjikian, S., Lejay, P., Manuel, P., Khalyavin, D., and Chapon, L. C. Anisotropy-Tuned Magnetic Order in Pyrochlore Iridates. *Phys. Rev. Lett.* **114**, 247202 (2015).

[4] Gardner, J. S., Gingras, M. J. P., and Greedan, J. E. Magnetic pyrochlore oxides. *Rev. Mod. Phys.* **82**, 53-107 (2010).

[5] Quilliam, J. A., Yaraskavitch, L. R., Dabkowska, H. A., Gaulin, B. D., and Kycia, J. B. Dynamics of the magnetic susceptibility deep in the Coulomb phase of the dipolar spin ice material $Ho_2Ti_2O_7$. *Phys. Rev. B* **83**, 094424 (2011).

[6] Jaubert, L. D. C., and Holdsworth, P. C. W. Signature of magnetic monopole and Dirac string dynamics in spin ice. *Nature Physics* **5**, 258-261 (2009).

[7] Jaubert, L. D. C., and Holdsworth, P. C. W. Magnetic monopole dynamics in spin ice. *J. Phys.: Condens. Matter* **23**, 164222 (2011).

[8] Castelnovo, C., Moessner, R. and Sondhi, S. L. Debye-Hückel theory for spin ice at low temperature. *Phys. Rev. B* **84**, 144435 (2011).

[9] Jaubert, L. D. C. Monopole Holes in a Partially Ordered Spin Liquid. *SPIN* **05**, 1540005 (2015).

[10] Brooks-Bartlett, M. E., Banks, S. T., Jaubert, L. D. C., Harman-Clarke, A., and Holdsworth, P. C. W. Magnetic-moment fragmentation and monopole crystallization. *Phys. Rev. X* **4**, 011007 (2014).

[11] Matsuhira, K., Hiroi, Z., Tayama, T., Takagi, S., and Sakakibara, T. A new macroscopically degenerate ground state in the spin ice compound $Dy_2Ti_2O_7$ under a magnetic field. *J. Phys. Condens. Matter* **14**, L559-L565 (2002).

[12] Tabata, Y., Kadowaki, H., Matsuhira, K., Hiroi, Z., Aso, N., Ressouche, E., and Fåk, B. Kagomé Ice State in the Dipolar Spin Ice $Dy_2Ti_2O_7$. *Phys. Rev. Lett.* **97**, 257205 (2006).

[13] Hiroi, Z., Matsuhira, K., and Ogata, M. Ferromagnetic Ising Spin Chains Emerging from the Spin Ice under Magnetic Field. *J. Phys. Soc. Jap.* **72**, 3045-3048 (2003).

[14] Fennell, T., Petrenko, O. A., Fåk, B., Gardner, J. S., Bramwell, S. T. and Ouladdiaf, B. Neutron scattering studies of the spin ices $Ho_2Ti_2O_7$ and $Dy_2Ti_2O_7$ in applied magnetic field. *Phys. Rev. B* **72**, 224411 (2005).
